# Multidimensional screening for predicting pain problems in adults: a systematic review of screening tools and validation studies

**DOI:** 10.1097/PR9.0000000000000775

**Published:** 2019-09-11

**Authors:** Elke Veirman, Dimitri M. L. Van Ryckeghem, Annick De Paepe, Olivia J. Kirtley, Geert Crombez

**Affiliations:** a Department of Experimental Clinical and Health Psychology, Faculty of Psychology and Educational Sciences, Ghent University, Ghent, Belgium; b Institute for Health and Behaviour, INSIDE, Faculty of Language and Literature, Humanities, Arts and Education, University of Luxembourg, Esch-sur-Alzette, Luxembourg; c Section Experimental Health Psychology, Clinical Psychological Science, Departments, Faculty of Psychology and Neuroscience, Maastricht University, Maastricht, the Netherlands; d Center for Contextual Psychiatry, Department of Neurosciences, KU Leuven, Leuven, Belgium

**Keywords:** Multidimensional screening, Yellow flags, Pain, Risk of bias

## Abstract

Screening tools allowing to predict poor pain outcomes are widely used. Often these screening tools contain psychosocial risk factors. This review (1) identifies multidimensional screening tools that include psychosocial risk factors for the development or maintenance of pain, pain-related distress, and pain-related disability across pain problems in adults, (2) evaluates the quality of the validation studies using Prediction model Risk Of Bias ASsessment Tool (PROBAST), and (3) synthesizes methodological concerns. We identified 32 articles, across 42 study samples, validating 7 screening tools. All tools were developed in the context of musculoskeletal pain, most often back pain, and aimed to predict the maintenance of pain or pain-related disability, not pain-related distress. Although more recent studies design, conduct, analyze, and report according to best practices in prognosis research, risk of bias was most often moderate. Common methodological concerns were identified, related to participant selection (eg, mixed populations), predictors (eg, predictors were administered differently to predictors in the development study), outcomes (eg, overlap between predictors and outcomes), sample size and participant flow (eg, unknown or inappropriate handling of missing data), and analysis (eg, wide variety of performance measures). Recommendations for future research are provided.

## 1. Introduction

Chronic pain is a common experience, with a prevalence of between 10% and 20% in the general adult population.^6,7,34,95,114^ Often, chronic pain is disabling and notoriously difficult to treat.^87^ At least 2 strategies are possible to face these challenges. First, we can develop new and better medical and psychosocial interventions.^19^ Second, we can prevent acute pain from becoming chronic. The latter requires an understanding of how and why acute pain becomes chronic, the identification of individuals at risk, and the timely delivery of preventive actions.^67,126^

Evidence has been accumulating that psychosocial variables are important in the prediction and prevention of chronic pain. First, available experimental and prospective research reveals the role of psychosocial factors in explaining pain, distress, and disability.^57^ The roles of learning, emotions, and cognitive factors are well established in laboratory studies,^123^ and a number of prospective studies have provided evidence for the role of psychosocial factors in the development and maintenance of pain.^3,60,102^ For example, Sobol-Kwapinska et al.^106^ reviewed predictors of acute postsurgical pain and found pain catastrophizing, optimism, expectation of pain, neuroticism, anxiety (state and trait), negative affect, and depression to be associated with acute postsurgical pain. Second, contemporary theoretical models have provided insight into how acute pain patients with a particular psychosocial profile may become stuck in a vicious cascade of further pain, distress, and disability.^13,122^ Third, evidence is increasing that the timely delivery of cognitive-behavioral interventions can prevent persistent disability.^67^

Taking this evidence into account, Kendall et al.^51^ called for the routine assessment of psychosocial factors in people experiencing acute pain. They introduced the concept of “yellow flags” as a method to screen for psychosocial risk factors predicting long-term disability, a concept that has been adopted by a growing number of researchers interested in examining the value of prognostic models.^26,27^ This has led to the development of screening tools that include various psychosocial risk factors and a recommendation for their use in clinical practice (eg, Keele STarT Back Screening Tool [STarT Back]^41^; Preventing the Inception of Chronic Pain [PICKUP]^115^).

Several reviews have summarized the predictive performance of screening tools.^30,42,49,68,99^ For instance, in a meta-analysis of screening tools, Karan et al.^49^ showed that screening tools poorly predicted pain, but were acceptable and excellent in predicting disability, and absenteeism, respectively (eg, STarT Back, OMPSQ). This meta-analysis is of high quality and according to the highest standards in the field.^71,109^ For that reason, our aim was not to focus upon the actual performance of the screening tools. Available meta-analyses^49,99^ have also noted that the methodological quality of studies investigating the predictive performance of screening tools is variable. Nevertheless, these reviews do not provide details of the methodological problems and limitations.

For that reason, our review focuses upon the methodological quality of studies that validate screening tools. First, a detailed analysis and synthesis of the methodological quality of studies is largely missing. Indeed, despite being considered fundamental to guide interpretation of findings, and recommendations for future research and practice,^52^ available reviews spend little or no attention to this topic. Second, the methodological quality of the studies in these reviews, typically described as “risk of bias,”^40^ was assessed using instruments that were not specifically designed for evaluating the quality of prediction models (eg, Quality in Prognostic Studies tool [QUIPS]).^36,37^ Recently, “Prediction model Risk Of Bias ASsessment Tool” (PROBAST), a tool for assessing the risk of bias and applicability of diagnostic and prognostic prediction model studies, has become available and used.^76,127–129^

The aim of this systematic review was 3-fold: (a) to identify available multidimensional screening tools that include psychosocial risk factors for poor pain outcomes (development or maintenance of pain, pain-related distress, and pain-related disability) across pain problems in adults, (b) to evaluate the quality of prospective studies validating these screening tools with up-to-date standards for clinical prediction models, and (c) to synthesize methodological concerns that may bias the predictive performance of these screening tools.

## 2. Methods

### 2.1. Literature search and eligibility criteria

The literature search comprised 4 steps. First, a search was performed for studies published in peer-reviewed journals across relevant electronic databases (MEDLINE, PsychINFO, and Web of Science) using the following terms in the title, key words, or abstract: screen* AND (*tool* OR *questionnaire*) AND *pain* AND *risk*. Screening of titles, key words, and abstracts allowed identification of screening tools and eligible studies. Second, a list of publications was sent to lead authors in the field of pain research to ask for any other available screening tools of which they were aware. Third, the reference lists of relevant systematic reviews were hand-searched for any articles that were not yielded by our other search methods. Finally, when only the development article for a tool fulfilling the inclusion criteria (see below) was identified in the search, a search was performed for additional articles that fulfilled the inclusion criteria by screening all publications that cited this development article.

The following eligibility criteria were used to identify screening tools for inclusion in this systematic review:

(1) The screening tool is a self-report questionnaire.
(2) The screening tool is multidimensional, containing at least 2 psychosocial risk factors. The report of somatic experiences such as pain, radiation, or other somatic complaints is not considered as psychosocial factors.
(3) The screening tool aims to predict the development (<3 months) or maintenance (≥3 months) of pain, pain-related distress, or pain-related disability.
(4) The screening tool is specifically developed in the context of pain and can target any type of pain (eg, neck pain and low back pain).
(5) The screening tool is a standalone instrument. Therefore, the tool should not consist of a battery of questionnaires, as is often the case for research purposes.
(6) The screening tool is validated in at least 1 independent study, ie, using data that were not used to develop the screening tool.

Six criteria (listed below) were used to select studies for inclusion. Some criteria were included to set a minimum quality (eg, criterion 1), whereas other criteria were applied to narrow the scope of the review (eg, criterion 2).

(1) The study is a full report published in a peer-reviewed scientific journal.
(2) The study includes an adult sample (the average age of the sample was older than 18 years).
(3) At baseline, the study includes patients experiencing no or (sub)acute pain (<3 months), without restriction in the type of pain experienced (eg, musculoskeletal pain, neuropathic pain, and postoperative pain). In line with the development studies of screening tools, we excluded studies involving only patients with chronic pain (≥3 months). Studies involving mixed samples with (sub)acute and chronic pain patients were included. However, when data for separate subsamples were reported, we only included the samples of interest.
(4) The study includes at least 1 screening tool, which is used in its original form. Some differences in translations, item order, and response scale are accepted. Shortened versions are considered different instruments.
(5) The study includes at least one of the following outcomes during outcome assessment (<2 years after baseline assessment): (a) Pain intensity or pain bothersomeness, assessed using a Visual Analogue Scale (VAS), a Numeric Rating Scale (NRS), a verbal rating scale, or a Likert scale; (b) pain-related disability including activity limitations (ie, difficulties in executing a task or an action such as the ability to walk, eat, shower, or dress) and participation restrictions (ie, problems relating to the involvement in life situations such as sick leave or days absent from work or return to work status) according to the International Classification of Functioning, Disability, and Health (ICF) framework.^130^ Assessment of these outcomes could be performed with (a subset of questions from) a self-report questionnaire, single questions, or data from existing registration systems; and (c) pain-related distress (eg, anxiety, fear, or low mood), assessed through self-report measures.
(6) The study is a prospective cohort study including patients presenting in primary, secondary, and tertiary health care settings.

Finally, studies were considered ineligible if they aimed to investigate the impact of stratified care (ie, targeted treatment to patient subgroups based on the results of the screening tool) or interventions that specifically targeted psychosocial risk factors (ie, cognitive behavioral therapy) or they consisted of a randomized control trial. We reasoned that the focus of these studies is on the evaluation of a (psychological) therapeutic intervention and not on the investigation of the predictive value of screening tools.

### 2.2. Data extraction and risk of bias assessment

The assessment of the quality of studies that validated the selected screening tools was based upon a prepublication version of the Prediction model study Risk Of Bias ASsessment Tool (PROBAST) (personal communication, January 2017, Dr. Robert Wolff). The PROBAST has been developed by the Cochrane Prognosis Methods Group using a Delphi process, in which 40 experts in the fields of prediction research and systematic review methodology participated.^129^ Its use is recommended by most recent guidelines for performing systematic reviews and meta-analyses of prediction model performance.^16^

Data extraction of eligible validation studies was conducted by E.V. and O.K. following a customized PROBAST template that was created for each of the 5 risk of bias assessment areas: (1) *participant selection*, (2) *predictors*, (3) *outcomes*, (4) *sample size and participant flow*, and (5) *analysis* (details can be retrieved from the authors upon request).^74^ Extracted data formed the basis for the risk of bias assessment, where signaling questions across those 5 important areas were rated as *yes*, *probably yes*, *probably no*, *no*, or *no information,* with *yes* indicating the absence of bias and *probably no* or *no* indicating the potential for bias.

For *participant selection*, elements judged were whether appropriate inclusion and exclusion criteria were used and whether patients had a similar state of health at enrollment. For *predictors*, questions considered were whether definition and assessment of predictors were similar across participants, and whether definition and assessment of predictors were similar compared with those of the development model. For *outcomes*, important elements judged were whether a valid outcome was used, whether predictors were excluded from the outcome definition, whether definition and assessment of outcomes were similar across participants, whether definition and assessment of outcomes were similar compared with those of the development model, and whether outcome assessment was blinded to predictor data. For *sample size and participants flow*, elements judged were whether a reasonable number of outcome events were available, whether the time interval between predictor and outcome assessment was appropriate, whether all enrolled participants were included in the analyses, and whether missing data occurred and participants with missing data were handled appropriately. Finally, for *analysis*, evaluated elements focused on whether relevant model performance measures were evaluated. Domains were subsequently rated as *high*, *moderate*, *low*, or *unclear* risk of bias. Risk of bias assessment labels were discussed and assigned upon agreement among team members (G.C., D.V.R., and E.V.).

## 3. Results

### 3.1. Study selection

The study selection process was guided by the Preferred Reporting Items for Systematic Reviews and Meta-Analyses guidelines (PRISMA),^71^ except for a preregistration of the review. Electronic databases were searched from the earliest record available on September 15, 2016, resulting in 1850 records. After removal of duplicate articles, 2 reviewers (J.C. and E.V.) independently screened a selection of the titles, key words, and abstracts for possible study inclusion. First screening resulted in 187 remaining references.

In the second step, full copies of articles were obtained (E.V.). Full-text reading of these articles resulted in exclusion of several tools for the following reasons (1) not being a screening tool (eg, “Amsterdam Preoperative Anxiety and Information Scale”),^70^ (2) the screening tool was not developed in the context of pain (eg, “Distress and Risk Assessment Method”),^65^ (3) the screening tool did not assess any psychosocial factors (eg, “London Fibromyalgia Epidemiology Study Screening Questionnaire”),^54^ and (4) the screening tool assessed only 1 psychosocial factor (eg, “Fear Avoidance Beliefs Questionnaire”).^93^

For 3 potentially eligible screening tools, items were not available in the literature and author contact yielded insufficient access to the tools' items (“Nijmegen Outcome of Lumbar Disc surgery Screening-instrument”^17^; “ABLE Presurgical Assessment Tool”^2^; and “Psychosocial Risk for Occupational Disability Scale”^100^).

Finally, a number of eligible screening tools for which items were available in the literature were not included in the current review as no independent validation studies were retrieved from the electronic database search nor through cited reference search of the development articles of the screening tools (ie, “Absenteeism Screening Questionnaire”^116^; “Back Disability Risk Questionnaire”^103,104^; “Optimal Screening for Prediction of Referral and Outcome cohort yellow flag assessment tool”^58^; “Pain Recovery Inventory of Concerns and Expectations”^105^; “Screening-Instrument zur Feststellung des Bedarfs an medizinisch-beruflich orientierter Rehabilitation”^112^; “Traumatic Injuries Distress Scale”^125^; and “Work and Health Questionnaire”^1^).

**Figure 1.**
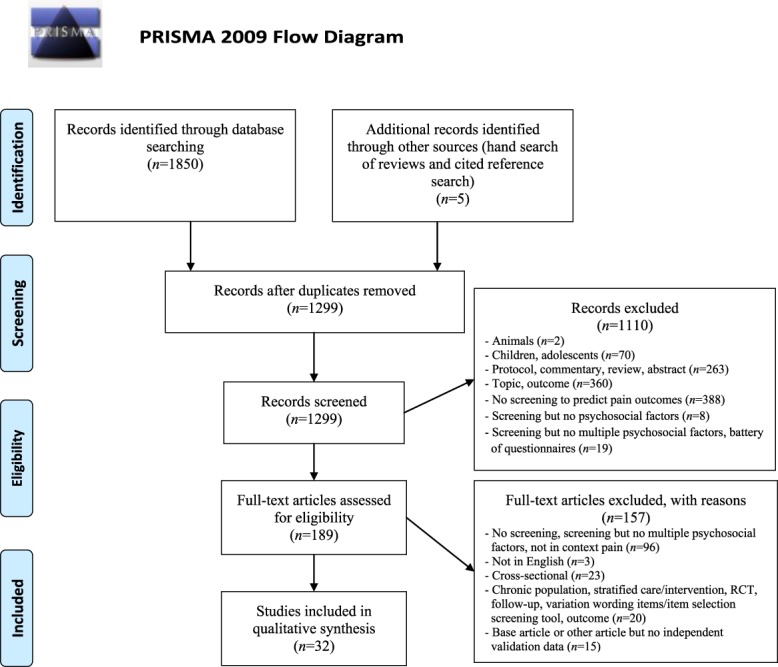
Flow of studies through the review.

In addition to the 27 articles that were considered eligible from the electronic database search, 2 articles^25,50^ were identified through cited reference search of the development articles of the screening tools on May 4, 2017, and 3 references^32,53,64^ were retrieved by hand-searching of relevant review articles^8,30,42,45,49,59,68,86,88,90,99,107^ (O.K.), resulting in a total of 32 references fulfilling the inclusion criteria for the current review. Additional author contact yielded no other tools or studies (see Figure 1 for a flowchart).

Doubts and disagreements on the inclusion of screening tools and eligible studies were resolved by discussion within the team (G.C., D.V.R., E.V., A.D.P., and O.K.) until consensus was reached. After finalizing the systematic search, all screening tools and development studies were retrieved to extract essential data for the risk of bias assessment. During the screening process, reviewers were not blind to authorship, institution, journal, or results.

### 3.2. Study characteristics: screening tools

The 32 included articles contained 42 study samples. Notably, several articles reported on a similar sample as earlier published articles, whereas other study samples completed multiple screening tools. The articles reported on the validation of 7 screening tools:

(1) Acute Low Back Pain Screening Questionnaire (ALBPSQ; 7 studies)^62^*/*Örebro Musculoskeletal Pain Screening Questionnaire (OMPSQ; 10 studies)^61^/Örebro Musculoskeletal Screening Questionnaire (OMSQ; 3 studies).^25^ The ALBSQ is a 24-item self-report questionnaire aiming to predict poor prognosis—operationalized as accumulated sick leave—in acute and subacute patients presenting with musculoskeletal pain (back, neck, and shoulder pain). A few years after its development, it was relabeled as the OMPSQ, including an additional unscored item on employment status. More recently, the OMSQ broadened the focus of the ALBPSQ to general musculoskeletal problems and simplified the questions.
(2) Örebro Musculoskeletal Pain Screening Questionnaire short version (OMPSQs; 2 studies).^63^ The OMPSQs is a 10-item self-report questionnaire designed to predict disability—operationalized as sick leave—in workers suffering from musculoskeletal pain (back pain).
(3) Örebro Musculoskeletal Screening Questionnaire short version (OMSQs; 1 study).^23^ The OMSQs is a 12-item self-report questionnaire aiming to predict a wide variety of outcomes—including problem severity, functional impairment, absenteeism, long-term absenteeism, cost, and recovery time—in acute and subacute work-injured patients presenting with musculoskeletal pain (whiplash, low back pain).
(4) Heidelberger Kurzfragebogen Rückenschmerz (HKF-R10; 1 study).^79^ The HKF-R10 is a 27-item self-report questionnaire developed to predict the likelihood of chronicity in patients with acute low back pain.
(5) Pain Belief Screening Instrument (PBSI; 1 study).^97^ The PBSI is a 7-item self-report questionnaire aiming to predict disability in subacute and chronic pain patients with musculoskeletal pain (neck, shoulder, and low back pain).
(6) Keele STarT Back Screening Tool (SBT; 11 studies).^41^ The SBT is a 9-item self-report questionnaire developed to predict poor outcome—operationalized as disability—in (sub)acute and chronic primary care patients with nonspecific low back pain.
(7) Preventing the Inception of Chronic Pain (PICKUP; 2 studies).^115^ The PICKUP is a 5-item self-report questionnaire aiming to predict the risk of chronic low back pain in patients with acute low back pain.

An overview of the included instruments and more detailed characteristics (as described in the base article) can be found in Table 1.

**Table 1:**
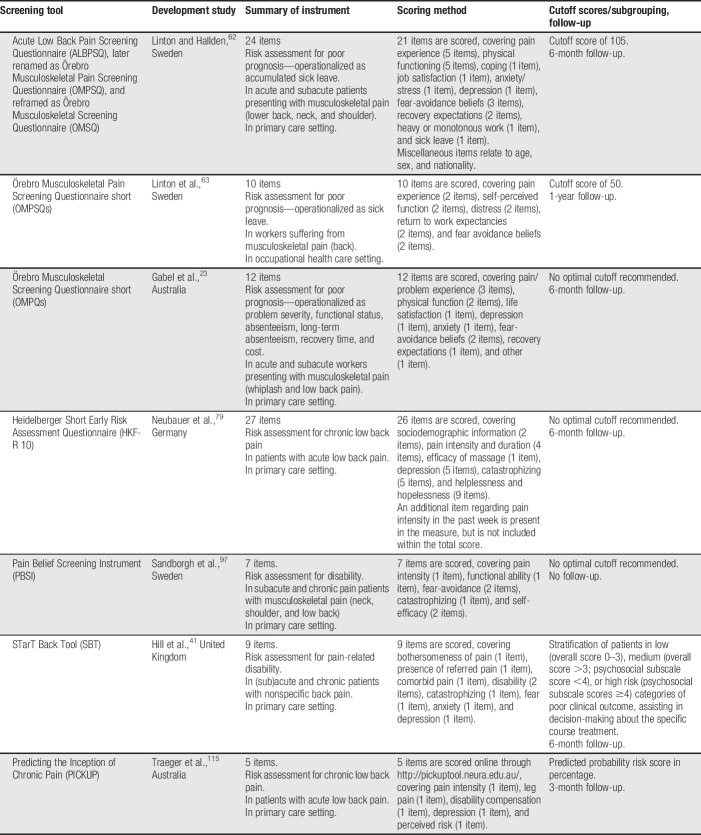
Summary of included screening tools.

### 3.3. Study characteristics: sources and samples

Studies were conducted between 2000 and 2017.^43,50^ The majority of the studies included samples that were collected in Northern European countries (N = 11) or Western European countries (N = 11). A small number of studies collected data from samples outside Europe, including Canada (N = 1), the United States (N = 3), Australia and New Zealand (N = 7), and China (N = 1).

Sex and age of participants differed largely between study samples. The average/median age of participants ranged between 37.7 years and 53.0 years.^21,64^ The sex of participants varied from 33.7% female participants to 83.0% female participants.^18,63^

Study samples were collected in primary care (83.3%) and secondary care settings (11.9%), and 1 study included a combined sample of participants from primary and secondary care units (4.8%).^92^ The terminology used to describe the settings varied, by reference to providers (eg, general practitioner or a physical therapist)^61^ and/or type of services (eg, spinal outpatient clinic).^50^ Although some studies detailed the treatment patients received (eg, work conditioning program),^66^ others often did not (eg, treated as usual).^81^ If information about the use of treatments is reported with insufficient detail, it can potentially bias performance results of the included screening tools because it does not allow researchers to evaluate the impact it might have had on the results.^82,83^ Moreover, within the studies that reported on the use of treatments, none of the studies accounted for treatment use.

Most study samples comprised participants with musculoskeletal pain. In particular, patients with back pain were overrepresented. Study samples often also included participants from other populations, such as those experiencing neck pain, pain between the shoulder blades,^124^ or multisite pain^24,25^ (see Table 2 for an overview).

**Table 2:**
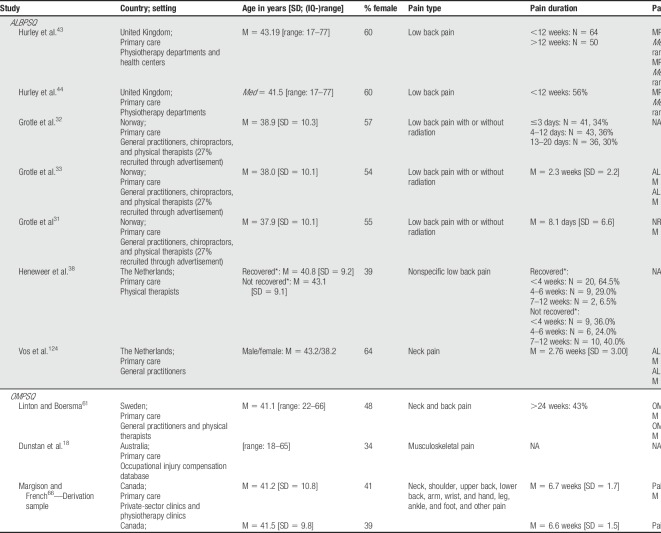

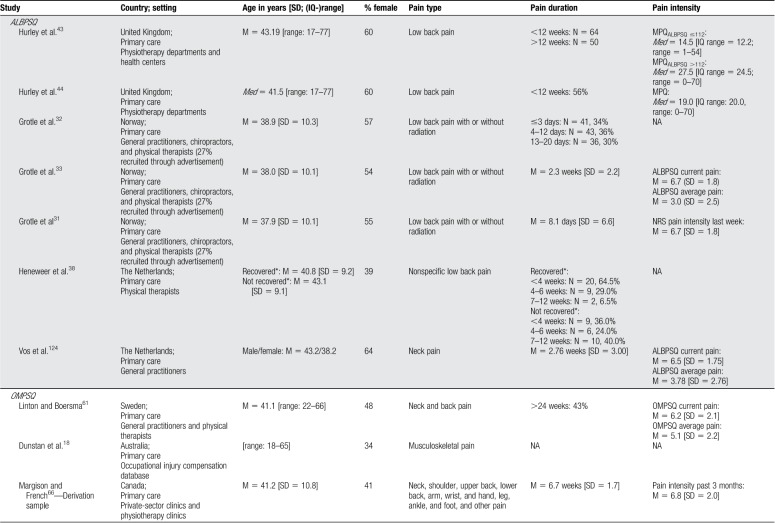

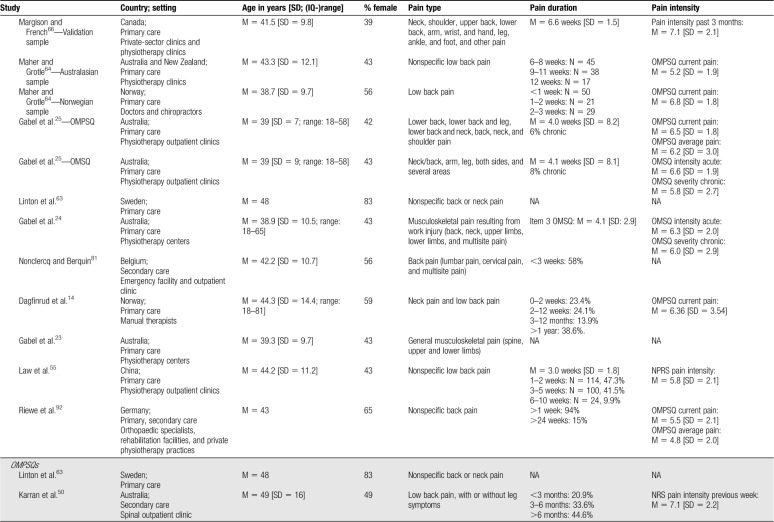

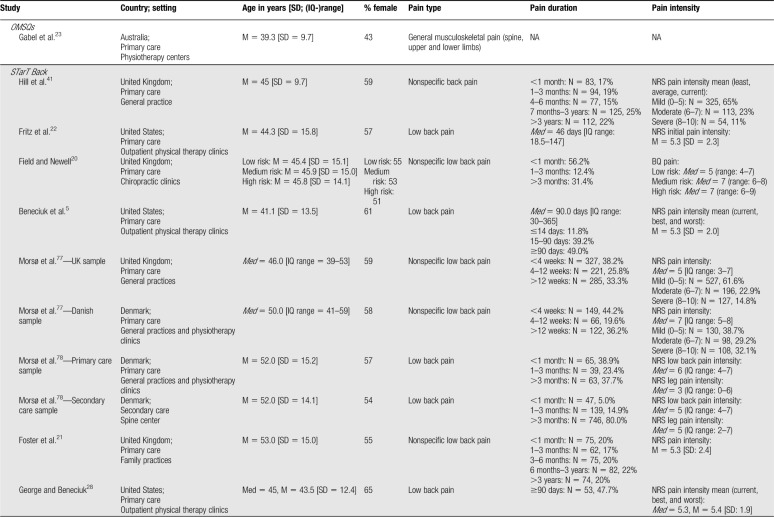

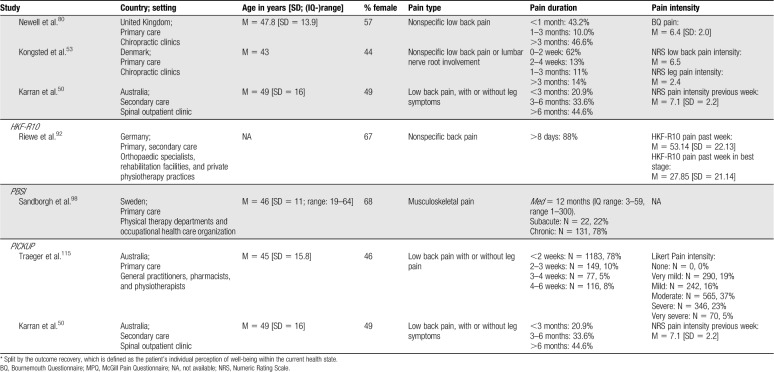
Key study and participant characteristics of included validation studies.

### 3.4. Risk of bias assessment of included studies

#### 3.4.1. Participant selection

The majority of the study samples consisted of mixed samples containing both acute and chronic pain patients (59.5%). The remaining samples comprised patients with acute pain (33.3%) or samples for which the type of pain (acute, subacute, or chronic pain) was not clearly described (7.1%) (Table 2).

For the PROBAST “participant selection” domain, the majority of the study samples were rated as having a moderate risk of bias (51.1%). Fewer study samples were rated as having low (16.7%) or high risk of bias (19.0%). For the remaining study samples, the risk of bias was rated as unclear (Table 3) because the presented information was insufficient to evaluate the appropriateness of the inclusion criteria or the state of health of participants. The reasons for increasing the risk of bias related to the specified inclusion and exclusion criteria and differences in the state of health of participants at enrollment.

**Table 3:**
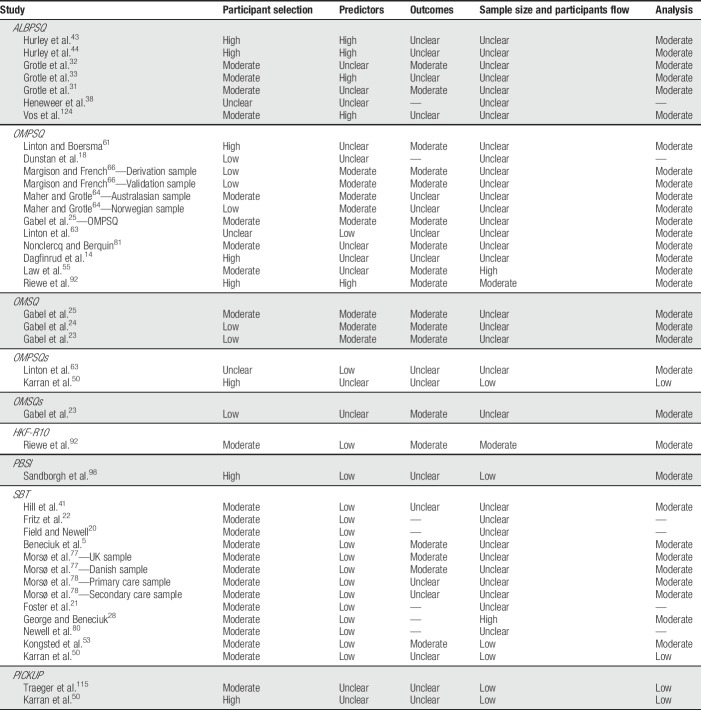
Methodological quality of included validation studies.

##### 3.4.1.1. Inclusion and exclusion criteria

The eligibility criteria were sometimes inappropriate or unclear. For example, some studies did not exclude unemployed participants^14^ or did not report information on employment,^38^ although the screening tools contained work-related questions. Most studies reported inclusion and exclusion criteria. However, sometimes the criteria had to be retrieved from descriptive information^66^ or from a previously published study.^64^

##### 3.4.1.2. Participants' state of health at enrollment

Although most studies aimed to recruit a homogeneous sample, other studies did not. Participants were found not to be in a similar state of health at baseline in cases when studies included patients with (sub)acute and chronic pain in a single sample.^5,14,20–22,25,28,41,43,44,50,53,61,63,64,77,78,80,92,98^ For instance, despite George and Beneciuk^28^ reported detailed information about their patients with (sub)acute and chronic pain, the analyses were based upon the full sample. For a considerable number of studies, the state of health of the participants had to be derived from descriptive information. For example, Margison and French^66^ only reported on average pain duration in weeks. Sometimes, insufficient information was available to conclude whether participants were in a similar state of health at enrolment^63^ (Table 2).

#### 3.4.2. Predictors

The most frequently used screening tool was the ALBPSQ/OMPSQ/OMSQ. However, we noted that the cutoff score to identify the high risk group varied substantially, ranging between 72 and 147 (Table 4).

**Table 4:**
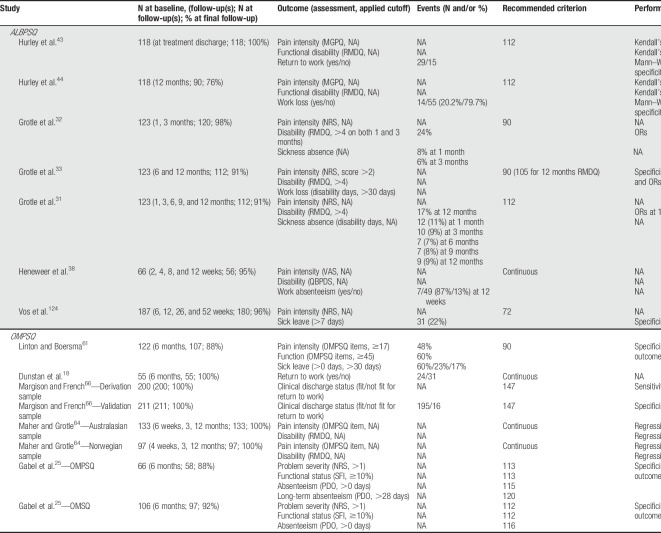

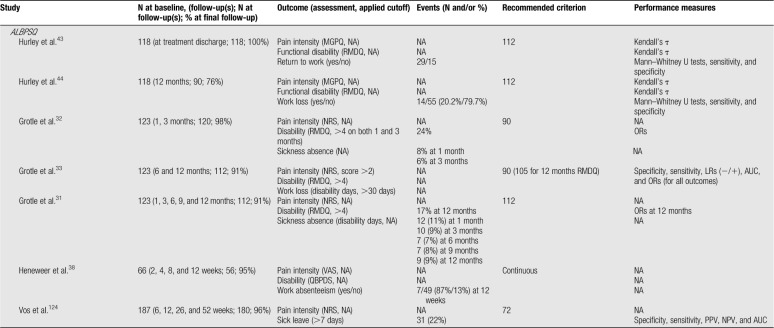

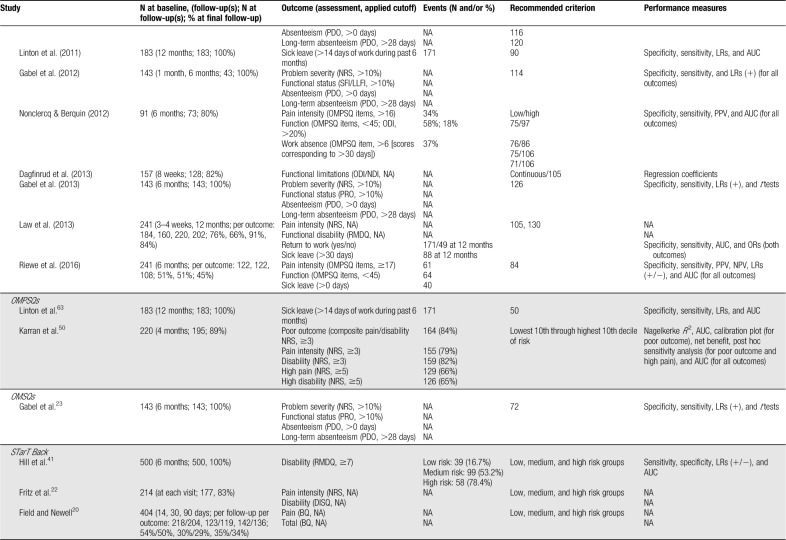

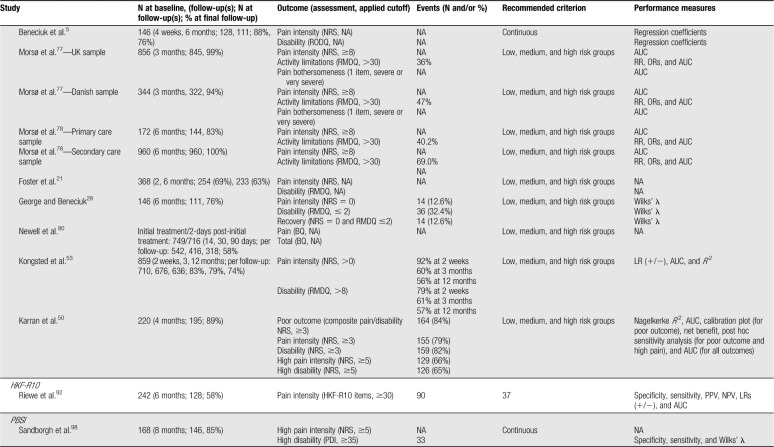

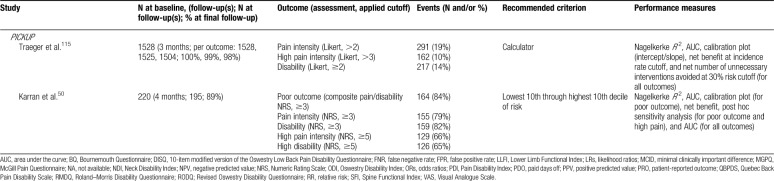
Key predictor, outcome, sample size and participants flow, and analysis characteristics of included validation studies.

For the PROBAST “predictors” domain, the majority of the study samples were rated as having a low risk of bias (40.5%). Only a small number of study samples were rated as having moderate (19.0%) or high risk of bias (11.9%). For 28.6% of the study samples, the risk of bias was rated as unclear (Table 3) because the presented information was insufficient to evaluate whether differences occurred in the assessment of the screening tools either across participants or compared with the development study. The reasons for increasing the risk of bias related to differences in the assessment of the screening tools across participants and differences in the assessment of screening tools compared with the development study.

##### 3.4.2.1. Definition and assessment of predictors across participants

Study samples that validated the ALBPSQ, the OMPSQ, and the PICKUP—tools that include work-related questions—sometimes did not report information on employment status. This could mean that participants were all employed, or that some of the participants were unemployed, but it was not reported.^50^ Furthermore, those studies that did report on employment status did not always administer these tools in a similar way across participants. For example, Hurley et al. instructed participants to fill out ALBPSQ work-related questions as best they could, even when they were unemployed.^43,44^ When these questions were left blank, the mean score of the other questions was used as replacement. In the study by Grotle et al.,^33^ it is noted that for participants who were unemployed, OMPSQ work-related questions were replaced by the mean score of the other questions.

##### 3.4.2.2. Definition and assessment of predictors compared with the developmental model

Furthermore, across the included studies, significant variation was observed in the applied screening tool cutoff points used to categorize patients. Selective reporting of results based only on cutoff values other than those specified in the original development study for the screening tool, was considered a risk for underestimation or overestimation of the screening tool's predictive accuracy. Moreover, variable use of cutoffs prohibits to estimate the influence of a given setting on the performance at the recommended (original) threshold. For example, for the ALBPSQ, the standard cutoff originates from Linton and Halldén,^62^ who used 105 as their cutoff score for detecting poor prognosis in the form of sick leave. Hurley et al.^44^ and Vos et al.^124^ only reported results using a cutoff of 112 and 72, respectively, for the outcome sick leave. In addition, few studies also treated screening tool scores as continuous without additional reporting of the cutoff values from the screening tool's development study^38^ (Table 4).

#### 3.4.3. Outcomes

The majority of study samples assessed one or more outcomes related to *pain* (66.7%), activity limitations (54.8%), and participation restrictions (50.0%). In addition, about half of the study samples reported also mixed or composite outcomes (40.5%) (Table 4).

For the PROBAST “outcomes” domain, the majority of the study samples were assigned an unclear risk of bias (40.5%), mainly due to insufficient information to evaluate blinding, or a moderate risk of bias (42.9%). None of the study samples were rated as having low risk of bias. For 16.7% of the study samples, the risk of bias was not rated because no performance measures were reported for the outcomes of interest (Table 3). The reasons for increasing the risk of bias related to the validity of the outcome, overlap between predictors and outcomes, differences in the assessment of outcomes across participants, differences in the assessment of outcomes compared with the development study, and blinding.

##### 3.4.3.1. Validity of outcome definition

Outcome measures that mixed outcome domains were rated as inadequate. Also, composite outcomes that combined outcome measures or outcome domains were considered inadequate.^28^ For example, the 10-item modified version of the Oswestry Disability Index contains items that assess activity limitations and participation restrictions.^22^ Mixed or composite outcomes have the potential to increase the event rate and thus the statistical power. However, they may be misleading when the outcome domains included in the outcome differ in importance to patients, the number of events in the outcome domains of greater importance is small, and the magnitude of effect differs markedly across the outcome domains.^72^

##### 3.4.3.2. Exclusion of predictors from outcome definition

Next, overlap between predictor and outcome assessment was frequently observed and considered as problematic. Several studies used items of the investigated screening tool, measured at follow-up, as primary outcome. For instance, Linton and Boersma^61^ used the OMPSQ in its entirety during the outcome assessment, selecting the items on pain, activity limitations, and sick leave. Studies also often included outcomes that showed overlap with domains assessed by the screening tool items. In the study by Grotle et al.,^32^ both the activity items of the ALBPSQ and the items of the Roland–Morris Disability Questionnaire (RMDQ) outcome measure address activity limitations. This overlap may lead to overestimation of the predictive performance of the screening tool.^91,117^

##### 3.4.3.3. Definition and assessment of outcomes across participants

For all studies, outcomes were defined and determined in a similar way across participants. However, they were not always defined and determined similarly to those in the development studies. Indeed, although different outcomes most probably have different predictors, a number of studies targeted outcome domains (eg, pain intensity through OMPSQ items and activity limitations through the RMDQ and not participation restrictions through accumulated sick leave)^64^ which differed from the development study. Other studies focused on similar outcome domains, but used other measures (eg, activity limitations through a NRS and not the RMDQ due to the large amount of missing data).^50^

##### 3.4.3.4. Definition and assessment of outcomes compared with the developmental model

In addition, some studies focused on similar outcome domains and used the same outcome measures as the development study, but used different cutoff points for the outcome measures from those used in the development study. For example, large differences were observed for sick leave. Vos et al.^124^ defined long-term sick leave as >7 days off work, while Linton and Hallden^62^ initially defined long-term sick leave as being sick listed for >30 days (Table 4).

##### 3.4.3.5. Determination of outcomes without knowledge of predictor information

Information on blinding was most often not reported, which could either mean that the outcome assessment was not blinded or that it was blinded but not described. In cases where studies reported on blinding of outcome assessment, researchers usually applied blinding.^24^

#### 3.4.4. Sample size and participant flow

There was a huge difference between sample sizes of the validation studies. Sample sizes varied considerably at follow-up, ranging from <100 participants,^18,25,38,43,44,64,81^ over 500 to 1000 participants,^41,53,77,78,80^ to >1500 participants.^115^ Also, the number of outcome events differed largely between studies ranging from 14 to 291. The most frequently observed time intervals were 3, 6, and 12 months^92^ (see Table 4 for an overview).

Few studies were rated as having low (16.7%), moderate (2.4%), or high (4.8%) risk of bias for the PROBAST “sample size and participants flow” domain. The majority of studies were assigned an unclear risk of bias (76.2%; Table 3) because insufficient information was presented to evaluate the number of outcome events, the inclusion of enrolled participants, or the occurrence and handling of missing data. The reasons for increasing the risk of bias related to the number of outcome events, the time interval between the assessment of the screening tools and the outcome assessment, dropout, and missing data.

##### 3.4.4.1. Number of outcome events

The number of events (ie, the number of individuals with the outcome event) was not reported in a large number of studies^5,14,21–25,33,38,64,66,80^ and considered inappropriate in 5 studies.^28,31,44,63,81^ These studies reported <20 events, raising the issue of overfitting (ie, the probability of an event is typically underestimated in low-risk patients and overestimated in high-risk patients).^4,85^

##### 3.4.4.2. Time interval between predictor assessment and outcome determination

Studies sometimes performed multiple follow-ups, reporting results on the predictive validity for one or only a selection of follow-ups (eg, follow-ups at 2- and 4-week intervals until discharge or study completion at 6 months, report of results for 6-month follow-up).^24^ Time between screening and outcome assessment was considered inappropriate when results only reported on follow-ups of <3 months, as chronic pain is defined as pain ≥3 months (eg, six weeks).^66^ Follow-ups >12 months were also considered inappropriate, as people's (mental) health status changes during the follow-up period and the baseline information becomes increasingly less accurate as time passes (none of the studies). In addition, follow-ups that varied across participants (eg, at treatment discharge, dependent on the number of therapy treatments)^43^ were deemed inappropriate. Surprisingly, most studies did not present any theoretical considerations underpinning the choice of a specific follow-up timeframe (Table 4).

##### 3.4.4.3. Inclusion of enrolled participants in analysis

Dropout attrition is often poorly reported or presented in a way that prevents readers from being able to fully understand the risk of attrition bias. Studies often limit themselves to reporting the dropout rate. We considered dropout as inappropriate when >20%^96^ of the participants were lost at follow-up.^5,20,21,28,44,53,80,92^ However, dropout can occur for a number of reasons that may lead to differential dropout, such as motivation (participants lost interest), mobility (participants moved and are no longer able to continue participation), morbidity (participants experience illness preventing their participation), or mortality (participants die before study completion). For example, a low psychosocial risk group may lose more unmotivated participants—that in turn may have different outcomes due to being unmotivated—than a high psychosocial risk assessment group, and this differential dropout may lead to differences in outcomes measured among the remaining participants. Reasons for dropout are, however, rarely specified among the included studies. Furthermore, although characteristics of dropout (ie, baseline characteristics: eg, age, sex, pain intensity, and pain duration) should be available to examine whether systematic differences exist between those who completed a study and those who dropped out,^36^ only few studies reported on the differences between completers and noncompleters.^5,28,44,50,53,80,81,98^ Of these studies, some provided a detailed tabulation of the characteristics and statistical comparison,^50^ whereas other studies only reported the characteristics for which differences were found.^5^ Further, numerous studies do not mention whether differences were examined, which could either mean that differences were examined for all or some baseline characteristics but none were found, or no differences were tested.^55^

##### 3.4.4.4. Handling of missing data

Finally, studies did often not report on missing values or how they were or would have been handled,^78^ which could either mean that there were no missing data or that missing data were present but not described. Missing values were considered inappropriately handled when complete-case analysis was applied.^92^ They were judged as appropriately handled when multiple imputation was used.^74^ For example, Karran et al.^50^ used Little's Missing Completely at Random test to determine whether values were missing completely at random and used a maximization algorithm to impute missing values.

#### 3.4.5. Analyses

Statistics of reported performance measures for pain and related outcomes varied widely. Many studies report sensitivity and specificity of screening tools,^61^ whereas others included further details, reporting area under the curve using receiver operating characteristics analyses.^53^ Wilk's lambda for discriminative validity is also reported in some studies,^28,98^ as are the odds ratios from logistic regression analyses^33^ (see Table 4 for an overview).

For the PROBAST “analyses” domain, the majority of study samples were assigned a moderate risk of bias (76%), and only a few study samples were rated as low risk of bias (9.5%). For 14.3% of the study samples, no risk of bias labels was assigned because no performance measures were reported for the outcomes of interest. The reason for increasing the risk of bias related to the poor use of the performance measures.

##### 3.4.5.1. Evaluation of relevant model performance measures

Statistical analyses were found appropriate when they reflected both calibration (ie, agreement between predicted and observed event rates) and discrimination (ie, the screening tool's ability to distinguish between patients developing and not developing the outcome of interest) components of predictive validity for pain and related outcomes.^74^ This was only the case in 2 studies.^50,115^ These studies also reported more recently introduced performance measures (eg, net benefit). Moreover, not all studies reported performance measures for pain and related outcomes despite assessing those outcomes. Some studies reported on the course of particular pain and related outcomes. For example, Grotle et al.^31^ reported the course of pain intensity, disability, and sickness absence from baseline across follow-ups, but reported no information on the predictive validity of the ALBPSQ for those outcomes, except for disability where odds ratios were provided. Other studies reported differences in mean scores on the screening tool for particular outcomes, used change scores for particular outcomes, or reported on composite outcomes. For example, Dunstan et al.^18^ reported differences in mean ALBPSQ scores between those who did and did not return to work. Dagfinrud et al.^14^ assessed functional limitations at baseline and follow-up; however, the predictive validity of the OMPSQ was examined for functional improvement, and the categorization of those that were improved and those that were not was based on change scores. Finally, George and Beneciuk^28^ assessed pain intensity and disability; yet, discriminative validity was only examined for recovery, a composite pain intensity and disability outcome. Still others assessed pain and related outcomes, but only reported performance measures related to outcomes that were not within the scope of the current review. For instance, Heneweer et al.^38^ assessed pain intensity, disability, work absenteeism, and self-reported recovery, but only reported area under the curve values for the ALBPSQ total and subscale scores in predicting recovery or nonrecovery at final follow-up (Table 4).

## 4. General discussion

This review (1) identified multidimensional screening tools that assess psychosocial risk factors for poor pain outcomes, (2) appraised the quality of the evidence in prospective studies validating these tools, and (3) synthesized common methodological concerns in these validation studies.

Seven screening tools were identified, all developed for use in primary care settings to predict chronic pain (HKF-R10, PICKUP) or chronic disability (ALBPSQ/OMPSQ, OMPSQs, OMSQs, PBSI, and STarT Back) in patients with back pain. Notably, we found no tools for the prediction of pain-related distress, a key indicator of health, or for the prediction of acute pain onset, including postoperative pain. These appear to be significant gaps in the literature.^101^

We assessed the quality of the evidence of 32 studies including 42 study samples aiming to validate the predictive value of identified screening tools. Overall, studies showed a moderate risk of bias, which varied largely from domain to domain. Here, we discuss the most notable methodological problems.

Most screening tools were developed to predict the chronification of pain problems, except for the SBT and the PBSI, which were developed to support decision-making for a wide range of patients with pain conditions, regardless of pain duration.^41,97^ It is reasonable to expect that validation studies include similar patient populations as those from the development study. Surprisingly, this was often not the case. Indeed, although most tools were developed to be used in patients with acute pain, a substantial number of these validation study samples included also patients with chronic pain. This is concerning for several reasons. First, these studies do not address the same key question as the development study. It may also well be that risk factors developing chronic pain are different from predictors for the maintenance of chronic pain. Second, it is likely that the recovery rate of chronic pain is less than the one of acute pain.^39^ Therefore, the presence of chronic patients with chronic pain may (at least partly) account for the apparently high performance in predicting poor pain outcomes. This complicates interpretation of results and may result in an underestimation or overestimation of the predictive value of the screening tools. There is a need to define the inclusion criteria for participants in a more clear and restrictive way and to align these with the original purpose of the screening tools.

The success of initial studies revealing the value of psychosocial risk factors in predicting chronic pain problems has boosted research in this area. However, some of the original studies were designed with specific (clinical) groups in mind. An example is the ALBPSQ, which was designed to target a working population. Some items that are directly related to work (eg, “If you take into consideration your work routines, management, salary, promotion possibilities, and workmates, how satisfied are you with your job?”) are therefore inapplicable to a nonworking population. The authors have addressed this problem in various ways. Some replaced the missing scores for those items by the mean for nonworking patients.^33^ Others asked patients to fill out those questions related to either current paid or unpaid work.^43,44^ Likewise, screening tools were developed for patients with musculoskeletal, in particular back pain, but studies have also investigated the value of the tools in other patient groups (eg, neck pain).^124^ Sometimes, items have been adapted accordingly and/or left out. There is a lack of evidence, however, to suggest that these changes are appropriate for the populations in question.

All studies agree that screening tools need to predict poor pain outcome. However, there is less agreement about what exactly poor outcome means. Indeed, a gold standard for poor outcome is lacking. The constructs addressed and the measures and cutoffs used vary largely between studies. For some, poor outcome simply means pain, for others not being able to work, or difficulties in performing physical activities. However, different outcomes most probably also have different predictors. The broad use of the umbrella term “disability” brings additional complications. Indeed, in pain research, “disability” may indicate difficulties in performing particular physical activities (eg, ability to walk, eat, shower, or dress) but also problems related to social role functioning (eg, sick leave, days absent from work, or return to work status). According to the International Classification of Functioning (ICF),^130^ these are 2 different constructs, ie, activity limitations and participation restrictions, which should not be confused. The lack of a gold standard may also explain the inconsistency in criteria used across studies. For instance, Morsø et al. defined poor pain outcome as a score greater than 7 on an 11-point NRS,^77,78^ whereas George and Beneciuk^28^ defined it as a score greater than 0. It is obvious that the patients defined as recovered differ between these studies. The use of an agreed-upon set of outcome measures may provide a solution.^10,11,89^ In doing so, we also recommend the selection of measures that are readily applicable to different contexts—occupational and nonoccupational settings—and to different pain problems. Such measures already exist, but are underused (eg, Patient-Reported Outcomes Measurement Information System, PROMIS,^9,118,119^ available at www.healthmeasures.net).

Some of the identified screening tools were developed to screen for psychosocial risk factors (“yellow flags”), or, at least, are presented as such in studies. Some cautionary notes are warranted. First, all screening tools also include items that could be categorized otherwise (eg, pain duration and disability compensation). Second, screening tools often contain items that could equally well be the primary outcomes (pain intensity, disability, and days off work). Although this may be less of a problem when simply aiming to predict, it is premature to explain the predictive power of these instruments in terms of psychosocial processes. Indeed, given that it is generally known that the best predictor of events in the future is their occurrence in the present or past, it remains to be investigated whether the predictive validity of screening tools is due to the overlap between predictor and outcome.^91,117^ To address this problem, one may examine whether tools are able to predict outcomes, beyond the predictive power of baseline pain and pain-related disability.

Most studies are not in line with the current guidelines for reporting measures of performance.^110,111^ In fact, there is a large disparity in reported performance measures. Many studies reported conventional performance measures, often reporting either calibration (ie, how close predictions are to observed outcomes) or discrimination (ie, screening tool's ability to correctly distinguish the 2 outcome classifications of event vs nonevent). However, the reporting of both performance measures is crucial. Furthermore, most studies do not consider the clinical consequences of decisions made using a screening tool. Therefore, there is the implicit assumption that false-positive (ie, patient being treated unnecessarily) and false-negative (ie, patient not getting a treatment that (s)he would benefit from) predictions are equally harmful (ie, equally weighted). More recent studies^50,115^ do consider the relative harms or benefits of these alternative clinical outcomes. They apply novel performance measures such as net benefit (ie, the expected utility of a decision to treat patients at some threshold, compared with a decision based on an alternative policy such as treating nobody)^75,110,111,120,121^ (see also www.decisioncurveanalysis.org).

An assessment of the risk of bias was not possible in a considerable number of studies because of incomplete reporting. A balanced evaluation of the risk of bias of studies may be impeded due to nontransparent reporting. An increased quality of reporting was observed over time, but there is still room for improvement and there is a need for guidance. The “Transparent Reporting of a multivariable prediction model for Individual Prognosis Or Diagnosis” (TRIPOD) statement is particularly helpful and provides guidance for the reporting of studies that develop, validate, or update prediction models^12,73^ (available atwww.tripod-statement.org). We encourage researchers to follow its recommendations. Equally important are the availability of study protocols and the availability of data sets. Protocol registration, either through publications, or through open science applications, may reduce the impact of publication bias.^84^ A large number of validation studies in our review reported significant results; yet, only 2 studies mentioned a protocol.^50,115^ Protocol registration may also reduce reporting bias.^40^ It is common practice to measure several outcomes, but the lack of a readily accessible research protocol makes these studies vulnerable to selective reporting of analyses that “worked.”^35^ Another possibility is to make data sets open, ie, available to all researchers.^115^ Available data sets provide the opportunity to conduct secondary analyses that may be informed by advances in theory and scientific standards in the field.

There are some limitations to our review. First, we used a strict search strategy. We excluded batteries of questionnaires and tools that were not originally developed in the context of pain. This may have resulted in missing instruments that are potentially valuable. For example, the Amsterdam Preoperative Anxiety and Information Scale (APAIS) was originally developed to evaluate patient's preoperative anxiety and need for preoperative information regarding the scheduled surgery and anesthesia.^70^ Subsequently, this tool was used to predict postoperative pain.^46,48^ Second, we focused upon multidimensional screening tools. Otherwise, one may make use of unidimensional questionnaires assessing single psychosocial risk factors to investigate the predictive power of unique psychosocial variables (eg, Pain Catastrophizing Scale^113^ and Tampa Scale for Kinesiophobia^69^) for poor pain outcomes. For screening purposes, however, one should aim to minimize the burden of filling out questionnaires for participants. The use of large questionnaire batteries should therefore be avoided. Third, this research field is quickly evolving, with new validation studies appearing at a fast pace. Since our search, new instruments have been validated in an independent study. For instance, the Optimal Screening for Prediction of Referral and Outcome cohort yellow flag assessment tool was developed in a cross-sectional cohort in 2016.^58^ Recently, a validation study was published.^29^ Fourth, clinical prediction modelling is a dynamic and evolving field^15,47,56,94,108–111^ (see also progress-partnership.org). One should keep in mind that the present review is an exploratory mapping of this rapidly evolving field. Assessment of the quality evidence in the included studies was based upon a prepublication version of the PROBAST. This version did not yet provide a guideline for scoring the questions. We constructed, therefore, our own coding system. Now, PROBAST has been published, with some minor changes from the prepublication version of the PROBAST (eg, the signaling questions of the domain “Sample size and participants flow” are now included in the domain Outcomes and the domain Analysis).^76,128^ Despite this minor changes, the resulting mapping fulfills the primary goal of providing an entry point to reduce risk of bias in this field. Fifth, we did not perform a meta-analysis. Several meta-analyses are available that synthesize the predictive value of screening tools. They indicate that (1) the predictive value of these screening is highly variable depending on the pain outcome of interest (eg, pain and disability) and (2) substantial heterogeneity between studies exist.^49,99^ Taking into account methodological differences and quality criteria is therefore crucial to further our understanding of the predictive value of screening tools. Our insights have the potential to improve research in this area and decision-making based on this research.

## Disclosures

The authors have no conflict of interest to declare.

Preparation of this article was supported by funding from the European Union's Horizon 2020 research and innovation program (Grant 633491).
